# Cognitive Gains from Gist Reasoning Training in Adolescents with Chronic-Stage Traumatic Brain Injury

**DOI:** 10.3389/fneur.2014.00087

**Published:** 2014-06-11

**Authors:** Lori G. Cook, Sandra B. Chapman, Alan C. Elliott, Nellie N. Evenson, Kami Vinton

**Affiliations:** ^1^Center for Brain Health, The University of Texas at Dallas, Dallas, TX, USA; ^2^School of Behavioral and Brain Sciences, The University of Texas at Dallas, Dallas, TX, USA; ^3^Department of Statistical Science, Southern Methodist University, Dallas, TX, USA

**Keywords:** adolescence, brain injury, cognitive plasticity, cognitive training, complex information, executive function, frontal lobe, reasoning

## Abstract

Adolescents with traumatic brain injury (TBI) typically demonstrate good recovery of previously acquired skills. However, higher-order and later emergent cognitive functions are often impaired and linked to poor outcomes in academic and social/behavioral domains. Few control trials exist that test cognitive treatment effectiveness at chronic recovery stages. The current pilot study compared the effects of two forms of cognitive training, gist reasoning (top-down) versus rote memory learning (bottom-up), on ability to abstract meanings, recall facts, and utilize core executive functions (i.e., working memory, inhibition) in 20 adolescents (ages 12–20) who were 6 months or longer post-TBI. Participants completed eight 45-min sessions over 1 month. After training, the gist reasoning group (*n* = 10) exhibited significant improvement in ability to abstract meanings and increased fact recall. This group also showed significant generalizations to untrained executive functions of working memory and inhibition. The memory training group (*n* = 10) failed to show significant gains in ability to abstract meaning or on other untrained specialized executive functions, although improved fact recall approached significance. These preliminary results suggest that relatively short-term training (6 h) utilizing a top-down reasoning approach is more effective than a bottom-up rote learning approach in achieving gains in higher-order cognitive abilities in adolescents at chronic stages of TBI. These findings need to be replicated in a larger study; nonetheless, the preliminary data suggest that traditional cognitive intervention schedules need to extend to later-stage training opportunities. Chronic-stage, higher-order cognitive trainings may serve to elevate levels of cognitive performance in adolescents with TBI.

## Introduction

Traumatic brain injury (TBI), defined as damage to the brain as a result of sudden trauma, is reported to be the most common cause of disability among youth in the United States today. The incidence of TBI peaks in adolescence, increasing from 600/100,000 at age 14 to more than 800/100,000 by 20 years of age (1). Considering the major developmental spurts in frontal brain regions during adolescence, combined with the real-life challenges faced by a teenager – whether it is heightened academic demands, social pressures, or introduction of complex tasks such as driving and job performance – this transition to adulthood is a pivotal time. Yet, in terms of brain development, teens are very much still “pediatric” (2–4), and adolescents with TBI may find this adaptation to growing demands even more challenging than their peers (5).

Neuroimaging investigations of the developing brain have advanced our understanding of the dynamic processes of myelination and synaptic pruning that occur during adolescence (2–4). In particular, longitudinal studies have demonstrated a protracted myelination of frontal networks continuing well into the mid 20s. Of relevance to pediatric TBI is that an injury during this lengthy developmental course may disrupt the maturation of frontal functions that support higher-order cognitive outcomes (6–8). These difficulties may ultimately manifest as a neurocognitive stall (9). A neurocognitive stall is illustrated when an individual seems to have recovered from his/her brain injury but begins to exhibit a plateau in cognitive performance relative to typical peers when evaluated years later. Essentially, many youth with TBI regain basic intellect and fundamental cognitive processes [e.g., Ref. (10)] but persistently struggle with more integrated, complex cognitive functioning [e.g., Ref. (11–15)]. Deficits may emerge in middle to upper grades, when many crucial frontal lobe functions are being called upon to navigate increased demands (5, 6, 9, 16, 17). Moreover, these emerging or persistent higher-order cognitive deficits have been linked to poor outcomes in school performance and social and behavioral functioning (5).

Unfortunately, few control trials exist that evaluate cognitive treatment effectiveness in TBI, especially at chronic recovery stages. A recent review of cognitive rehabilitation therapies for TBI conducted by the Institute of Medicine [IOM; Ref. (18)] highlighted the need for evidence-based treatments addressing higher-order cognitive deficits. Although several studies have reported positive effects of cognitive interventions for specific executive function skills [e.g., Ref. (19)], there is limited evidence of generalization of gains to untrained cognitive tasks. Additionally, the IOM report noted the paucity of evidence indicating improved patient-centered outcomes and “real-life” functioning (18).

In an effort to address this critical gap, the current exploratory study examined the effects of two forms of cognitive training, including training of gist reasoning versus training of rote memory learning, on ability to abstract meaning and recall details in adolescents with cognitive deficit due to chronic TBI. As highlighted below, this study was motivated by evidence of persistent residual impairments after moderate to severe TBI in the ability to synthesize or abstract/gist meanings from complex information, including a variety of information modalities (written, visual, and verbal content) [e.g., Ref. (12, 13, 15)]. The secondary aim was to explore whether either training was associated with generalized benefits to untrained domains, specifically frontally mediated measures of executive control.

The primary cognitive training in the current study targeted gist reasoning, the ability to strategically comprehend and convey generalized, core meaning(s) from complex information (20–24). The ability to construct abstract meanings rather than simply giving back information at a verbatim level has been shown to enhance long-term learning (21, 23, 25). Gist reasoning is critical for advanced learning and is a useful metric of cognitive health (22, 26) and has been associated with performance on executive control measures and everyday life functionality (11–13, 15, 27). For instance, the ability to process and recall the gist from classroom readings is related to depth and efficiency of learning compared to fact or detail-level recall in children (28). In order to target strengthening of gist reasoning skills, Chapman and colleagues developed Strategic Memory Advanced Reasoning Training (SMART), a program which promotes deeper understanding of information encountered in everyday life (27, 29–32).

The efficacy of the SMART program in improving gist reasoning abilities for young adolescents was investigated in a randomized control study at a low-income middle school (30). Students were randomized into three groups, receiving either SMART, a rote memory training, or information about the teen brain. The results indicated that the students who received SMART significantly improved in both gist reasoning and ability to recall facts. Those who received the memory training improved in fact-learning ability, but gist reasoning did not show significant gains. The third, information-based control group, failed to demonstrate significant gains in either domain. Generalized academic benefits were evidenced by performance on statewide achievement tests, which revealed significant gains in critical reasoning for SMART-trained students as compared to their peers who did not receive SMART.

Recent research extended investigation of SMART to a TBI population, namely, adults at chronic stages post-injury. In their randomized control trial, Vas et al. (27) examined the effects of SMART versus an information-based, new learning control in adults at least 1 year post-injury. The results identified significant gains in the SMART-trained group in abstracting meaning as compared to those who engaged in the bottom-up (i.e., non-strategic, information gathering) control group. Additionally, the benefits of SMART extended to untrained aspects of immediate memory, non-verbal reasoning, executive functions of working memory, inhibition, and cognitive switching, and improvements in daily functional activities (27). Further, the gains were maintained in the SMART-trained participants at 6-months post-training. Perhaps a more important finding was that significant gains occurred in reported daily-life domains of social abilities, work productivity, home management, and general well-being (27, 33).

To date, no known studies have investigated the effectiveness of gist reasoning training in adolescents with chronic-stage TBI. Therefore, the current study fills a void by providing preliminary evidence of training benefits in this high-incidence clinical population at a crucial stage of development. Based on the evidence summarized above, we proposed that gist reasoning training (SMART) would not only enhance performance on the primary trained domain of abstracting meaning from complex information (top-down ability), but also improve the ability to recall facts/details (bottom-up ability), an untrained aspect. The memory training was postulated to enhance only the ability to recall details, the primary trained domain. Moreover, we predicted that the effects of SMART would generalize to measures of executive control that were not specifically trained, including direct measures of working memory, inhibition, and non-verbal reasoning, as well as a parent-reported measure of real-life executive behaviors. No similar spillover effects were predicted with the memory training.

## Materials and Methods

### Participants

The study included 20 adolescents (ages 12–20) who had sustained a mild, moderate, or severe closed head TBI at least 6 months prior. They also had to demonstrate a gist processing deficit, as determined by below average baseline performance on the Test of Strategic Learning [TOSL; Ref. (34)]. Participants were pseudo-randomized into either the gist reasoning training group (*n* = 10) or the memory training group (*n* = 10).

Severe TBI was defined by lowest post-resuscitation Glasgow Coma Scale [GCS; Ref. (35)] score of 8 or below, moderate TBI as GCS of 9–12, and mild as GCS of 13–15 with persistent cognitive deficit. Exclusionary criteria were based on medical records and parental (or guardian) interviews prior to enrollment and included: previous hospitalization for head injury; pre-existing neurological disorder associated with cerebral dysfunction and/or cognitive deficit; previously diagnosed learning disability; pre-existing severe psychiatric disorder (e.g., schizophrenia, autism); history of child abuse; penetrating gunshot wound to the brain; history of hypoxia/anoxia; not an English language learner. Given its high prevalence in the TBI population, attention deficit disorder was not an exclusionary factor. See Table 1 for group demographic and injury characteristics. Also, see Table 3 for performance of participants in each group on baseline neuropsychological measures.

**Table 1:** **Demographic and injury characteristics of study participants**.

Variable	SMART group (*n* = 10)			Memory group (*n* = 10)		
	Mean	SD	Range	Mean	SD	Range
Age at test (years)	15.4	2.3	13–20	15.2	2.3	12–20
Age at injury (years)	10.5	3.6	4–15	12.2	3.3	7–17
Time post-injury (years)	4.9	4.1	1.0–13.1	3.2	2.2	0.6–6.0
Gender	6 Male; 4 female			7 Male; 3 female		
Ethnicity	5 Caucasian; 3 Hispanic			7 Caucasian; 3 Hispanic		
	2 African American			0 African American		
Injury severity	3 Severe; 1 moderate; 6 mild			4 Severe; 2 moderate; 4 mild		
Mechanism of injury	3 MVC; 2 struck by car;			2 MVC; 2 struck by car;		
	2 Falls; 1 ATV; 2 sports			2 Falls; 1 ATV; 3 sports		

*MVC, motor vehicle collision; ATV, all-terrain vehicle*.

Participants were recruited from cohorts in previous and ongoing pediatric brain injury studies at the Center for Brain Health of the University of Texas at Dallas and supplemented by referrals from the local Dallas/Fort Worth community. Written consent was obtained from the parent or legal guardian as well as written assent from the child for all study participants in approval and accordance with the guidelines of the institutional review board of the University of Texas at Dallas.

### Procedure

The current study was a single-blinded randomized control pilot, with participants being assigned to one of the two cognitive training protocols: (a) gist-based SMART or (b) fact-based Memory training. Participants were informed that the goal of the study was to compare the effects of two different training programs that could be beneficial to individuals with TBI. Outcome measures for both groups included the same battery of experimental and standardized cognitive tests, which were administered at baseline (pre-training) and upon completion of the training sessions.

### Outcome measures

#### Test of Strategic Learning

The primary outcomes were evaluated using the TOSL (34), which is used to identify how an individual understands and abstracts gist meanings from complex information. The TOSL is a criterion-referenced assessment, which evaluates skills encompassing different levels of complexity. The TOSL has been previously validated as a measure of ability to abstract meaning from complex information in typically developing youth (32, 36), in healthy adults (29), and in adults with TBI (27), and has demonstrated test–retest reliability (30–32). The TOSL has been shown to be sensitive to both gist-based and fact-based processing deficits in children with TBI (12, 13, 15), children with ADHD (37), and adolescents from poverty (30).

The TOSL is comprised of three separate texts of increasing length (ranging from 291 to 575 words) and complexity. Each text revolves around content typical to that encountered in everyday life or in classroom curricula. Each text includes three measurements. Two measurements evaluate ability to abstract generalized meanings through: (a) summarization and (b) one-sentence interpretation. Both measure high-level reasoning abilities to abstract the central message and to glean interpretations. The third measure assesses ability to recall the important facts through probes for each text. The child is first provided with an introductory example summary, including explanation of what aspects comprise a good summary. Then, the examiner reads aloud the text with a written copy from which the child follows along. Subsequently, the child is asked to provide a condensed version of the content (i.e., shortened in his/her own words), being sure to convey big, high-level ideas. The child is also asked to provide any messages or interpretations about life that can be gleaned from the information. Next, the child is given a series of eight probe questions to assess recall of important details for each text. The procedures are repeated for a total of three separate texts. Written responses were elicited for summaries and interpretative statements for all but four study participants (due to physical constraints), for whom oral responses were audio-recorded and transcribed for later scoring.

Test of Strategic Learning summaries, interpretations, and fact responses to probes were scored to generate three separate outcome measures. From the summaries, scores were generated based on inclusion of gist ideas (i.e., abstracting meaning). Specifically, points were awarded based on inclusion of gist ideas for all three texts, for a possible total of 26 points. The interpretive statements provided by the child were scored on a scale of 0–6 based on the level of abstraction represented and summed across all three texts, for a possible total of 18 points. Finally, the responses to each probe question were scored on a scale of 0–2 based on the accuracy and completeness of the response and summed across all three texts, yielding a possible total of 48 points.

Two trained raters independently score the TOSL summaries, interpretive statements, and responses to probes, including a rater blinded to participant, group, and assessment point (pre- versus post-training). The inter-rater reliability for the TOSL is 92% for gist-based scores and 98.5% for correctness of responses to probes in pediatric TBI patients (15). Disagreements between raters are resolved through discussion and mutual consensus.

#### General intelligence measure

An estimate of general intellectual ability was obtained from the two-subtest form of the *Wechsler Abbreviated Scales of Intelligence* [WASI; Ref. (38)], comprised of the Vocabulary and Matrix Reasoning subtests. For the Vocabulary subtest (a measure of verbal intelligence), the participant was asked to provide definitions for up to 38 progressively more difficult vocabulary words. For The Matrix Reasoning subtest (a measure of non-verbal intelligence), the participant was asked to provide answers to a visually presented multiple choice question (i.e., identifying one of five response options to complete a matrix) for examining pattern completion, classification, and analog and serial reasoning. *T*-scores were calculated for each subtest, as well as an estimated Full-Scale intelligence quotient (IQ) value for each study participant.

#### Executive function measures

Two subtests from the *Wechsler Intelligence Scale for Children* [WISC-IV; Ref. (39)] or the *Wechsler Adult Intelligence Scale* [WAIS-III; Ref. (40)] were administered to assess working memory, including the Digit Span and Letter–Number Sequencing subtests. For the Digit Span subtest, the participant was presented with oral sequences of numbers, which he or she was instructed to repeat aloud. The first condition elicited exact recall (i.e., in the same order), whereas the second condition elicited recall in reverse order. For the Letter–Number Sequencing subtest, the participant was presented with oral sequences of letters and numbers, which he or she was instructed to repeat aloud, organized by numbers first (in ascending order) and letters next (in alphabetical order). Scaled scores were calculated for each subtest for each participant and summed for analyses.

One subtest from the *Delis–Kaplan Executive Function System* [D–KEFS; Ref. (41)] was administered to assess inhibition abilities, namely, the Color–Word Interference subtest. This subtest examines the ability to inhibit an overlearned verbal response (i.e., reading printed words) in favor of naming the dissonant ink colors in which words are printed. Scaled scores were generated for each participant according to the total number of errors made on the inhibition condition.

#### Real-life functioning measure

The *Behavior Rating Inventory of Executive Function* [BRIEF; Ref. (42)], an 86-item questionnaire, was completed by a parent or legal guardian for each child. The BRIEF is designed to assess executive functions by means of rating children’s everyday behaviors (43, 44) in domains of inhibition, shifting, emotional control, initiating, working memory, planning/organizing, and self-monitoring. Responses were totaled to generate a standardized Global Executive Composite (GEC) for each participant, with higher scores indicating greater degrees of difficulty with real-life executive functioning behaviors.

### Cognitive training protocols

Both the gist reasoning training (SMART) and the Memory training were delivered individually. The trainings were each comprised of eight 45-min sessions administered over approximately 1 month. For both trainings, the instruction was hierarchical and dynamically interdependent, with each session building upon previous ones. Reinforcement and repeated practice of the preceding stages were given within each subsequent session. To promote reproducibility and fidelity, a project manual was utilized for both training programs. Current practice entails having an interventionist complete approximately 30 h of in-person training in order to be considered qualified to conduct each program. The trainings for all participants in this pilot study were administered by the first author, a trained doctoral level speech-language clinician, and were conducted one-on-one in the child’s own community (e.g., in the home, at the child’s school library, at the local community center). Both trainings utilized predominantly text-based materials as well as some of the child’s own schoolwork for immediate application and reinforcement when possible. The texts used represent middle- and high-school-level content that is typically encountered in language arts, history, and science coursework. Additionally, in both trainings, learned strategies could be applied across academic subjects.

#### Gist reasoning training – strategic memory advanced reasoning training

The SMART program was developed to train individuals to derive a deeper level of understanding by abstracting gist meanings from complex information. The SMART program is based upon cognitive neuroscience research explicating higher-order, top-down (i.e., strategy-based) cognitive processes (12, 13, 20, 28, 45–48). The core strategies are directed hierarchically and are explained and practiced through individual exercises and pen and paper activities in a student instructional manual. The strategies are designed to reinforce metacognitive processes that underlie reasoning and higher-order abstraction of multiple meanings (20, 46, 49–53).

The SMART program is distinct from other cognitive trainings in that it facilitates constructing abstracted/gist meanings through reasoning. To transform literal meanings into more global gist meanings calls upon integration of multiple cognitive processes such as inhibition, inferencing, paraphrasing, abstraction, and generalization (12, 13, 46, 49, 50, 54) rather than targeting specific processes in isolation. Skills addressed included higher-level cognitive strategies such as eliminating unimportant information (i.e., strategic attention), abstracting information in one’s own words (i.e., integrated reasoning), generating multiple interpretations and perspectives (i.e., elaborated reasoning), coming up with the personally applicable “take-home” messages, and applying new learning to create novel individually relevant ideas (i.e., innovation). From the first strategy introduced up to the final stages, the participant is increasingly challenged to employ top-down strategies of gist reasoning during learning rather than the bottom-up approach of verbatim information recall. Emphasis is also put on application of gist reasoning to other contexts/modalities (e.g., social scenarios, television or movie-viewing, etc.). See Table 2 for a description of each strategy in the SMART training sequence.

**Table 2:** **Outline of SMART and memory trainings**.

Process	Description	Session
**SMART TRAINING**		
Inhibiting	To delete/inhibit unimportant or irrelevant details	1
Organizing and managing	To organize and manage information by chunking similar ideas together	
Inferencing	To use inferencing to extract the deeper or more abstract meaning of information	2
Paraphrasing	To convey information in one’s own words	3
Synthesizing	To combine details together into gist-based concepts, using inferencing and paraphrasing	4
Integrating	To integrate previous knowledge with new information to formulate “take-home messages” from multiple perspectives	5
Abstracting and generalizing	To summarize using abstract, high-level gist-based concepts and applying learning beyond the immediate context to other contexts and situations	6–8
**MEMORY TRAINING**		
Attending	To focus attention on key details to remember by underlining	1
Rehearsing	To repeat/rehearse information to strengthen encoding	2
Retrieving	To practice retrieving the information from memory through self-test tools (text fill-in-the-blank, content questions)	3
Labeling	To use verbal mnemonics to improve efficiency	4
Visualizing	To use visual association strategies such as method of loci to facilitate recall	5–6
Using aids	To put important information on flash cards as a study and organization aid	7
Applying	To apply the different memory strategies together with schoolwork	8

#### Memory training

The Memory training was modeled after the classroom-based training instituted by Gamino et al. (30) with typically developing adolescents. The tools trained were based on cognitive neuroscience research describing the basic properties of bottom-up memory processes important for improvement of memorization skills. The materials used for the memory training imitated those used in the SMART program, including the use of many of the same texts and the presentation of activities in a student manual with a similar format. The memory training was comprised of both direct instruction regarding basic memory aids as well as opportunities to practice the processes. The memory techniques presented and practiced with pen and paper tasks included rehearsal (55), retrieval practice (56), association (57), and method of loci (58). Participants practiced using memory aids such as mnemonics, visualization (59), and flash cards. Through practice, the participants were expected to learn to use these rote memorization tools for verbatim recall of fact-based information. See Table 2 for a description of each strategy in the Memory training sequence.

### Analyses

At baseline, independent sample *t*-tests and Chi-square tests for independence were performed to establish comparability of the two training groups on demographic and baseline performance variables. To examine change between baseline and post-training assessments, paired *t*-tests were performed for each group. Due to this being a small-sample pilot study rather than an established clinical trial, Bonferroni alpha adjustments for multiple comparisons were not feasible for these initial exploratory analyses. However, separate analyses of covariance (ANCOVA) with *post hoc* Scheffe correction were also performed to determine the effect of group in order to ascertain differences post-training on performance variables. Also, effect sizes were calculated using Cohen’s *d* (60) and are reported to inform the strength of the observed effects according to the following guidelines: small (*d* of 0.2 or lower), medium (*d* of around 0.5), and large (*d* of 0.8 or higher). The small-sample size also did not allow inclusion in the model of other relevant variables, such as age and severity of injury, although, as noted below, the groups were closely equated for demographic and baseline cognitive performance variables.

## Results

### Baseline analyses

#### Demographics

Independent sample *t*-tests indicated no significant differences between the two training groups on variables of age at test [*t*(18) = 0.1936, *p* = 0.849], age at injury [*t*(18) = 1.0971, *p* = 0.287], or time post-injury [*t*(18) = 1.1717, *p* = 0.257]. Chi-square tests for independence also indicated no significant associations between group and gender [*X*^2^(1, *n* = 20) = 0.22, *p* = 0.639], ethnicity [*X*^2^(2, *n* = 20) = 2.33, *p* = 0.311], injury severity [*X*^2^(2, *n* = 20) = 0.876, *p* = 0.645], or mechanism of injury [*X*^2^(4, *n* = 20) = 0.40, *p* = 0.982] variables.

#### Primary outcome measure: Test of Strategic Learning

Table 3 presents the baseline and post-training scores by group for the performance measures. Prior to training, independent sample *t*-tests revealed no significant differences between the two groups on the baseline TOSL measures. This included measures of abstracting meaning (i.e., construction of gist ideas) [*t*(18) = 0.0000, *p* = 1.000], providing interpretative statements [*t*(18) = 0.7035, *p* = 0.491], and responses to probe questions regarding recall of text details [*t*(17) = 0.2276, *p* = 0.823].

**Table 3:** **Baseline and post-training outcomes by group**.

Variable	Group	Baseline *M* (SD)	Post-training *M* (SD)
**TOSL**			
Abstracted meanings	SMART	7.30 (4.83)	10.60 (4.93)
	Memory	7.30 (5.42)	7.90 (4.31)
Interpretation statement	SMART	13.00 (3.30)	15.00 (2.00)
	Memory	11.60 (5.36)	13.30 (4.90)
Recall of facts	SMART	35.70 (8.25)	40.30 (2.95)
	Memory	34.67 (11.45)	37.89 (6.88)
**GENERAL INTELLIGENCE**			
FSIQ	SMART	99.50 (8.38)	104.50 (6.88)
	Memory	96.20 (16.00)	98.00 (16.19)
**WORKING MEMORY**			
Digit span	SMART	8.89 (2.85)	10.11 (2.26)
	Memory	7.60 (2.46)	7.80 (2.90)
Letter–number	SMART	8.89 (2.71)	10.11 (1.96)
	Memory	7.10 (3.63)	8.00 (4.29)
Sum of scaled scores	SMART	17.78 (4.28)	20.22 (3.73)
	Memory	14.70 (5.68)	15.80 (6.65)
**INHIBITION – COLOR–WORD**			
Total errors scaled score	SMART	8.50 (2.27)	11.00 (1.76)
	Memory	7.10 (3.28)	8.80 (1.81)
BRIEF – PARENT FORM*			
GEC	SMART	63.13 (9.00)	57.50 (12.06)
	Memory	64.56 (11.97)	58.22 (13.33)

*For the BRIEF, lower score indicates fewer difficulties observed.

#### Secondary outcome measures

Prior to training, independent sample *t*-tests also indicated no significant differences between the two groups on the other cognitive measures administered. Namely, groups were comparable on measures of full-scale IQ [*t*(18) = 0.5778, *p* = 0.571], working memory (sum of scaled scores for Digit Span and Letter–Number Sequencing subtests) [*t*(17) = 1.3112, *p* = 0.207], and inhibition (Color–Word Interference total errors scaled score) [*t*(18) = 1.1091, *p* = 0.282]. The two groups were also comparable prior to training on the real-life executive function parent questionnaire (BRIEF GEC) [*t*(18) = 0.4448, *p* = 0.662].

### Post-training outcomes

#### Primary outcome measure: Test of Strategic Learning

To test the hypotheses that ability to abstract meaning (i.e., convey gist meanings) would change between baseline and post-intervention assessments, a paired *t*-test for each group was performed. Results indicated that there was a statistically significant increase in scores for abstracting meaning in the gist-based SMART group [*t*(9) = −5.906, *p* = 0.0002, *d* = 1.868]. This primary finding, although preliminary in nature, corresponds with a large effect size and, further, would hold up to a more stringent clinical trial-level Bonferroni alpha adjustment for the primary outcome measure (*p* < α/3 of 0.0167). In contrast, the analyses failed to reveal a significant change in abstracting meaning for the Memory training group [*t*(9) = −0.818, *p* = 0.434, *d* = 0.259]. To determine the difference between the groups on abstract reasoning ability post-training, an ANCOVA with *post hoc* Scheffe correction was performed. Results indicated that there was a significant effect of group [*F*(1,20) = 9.98, *p* = 0.006, *d* = 1.413], with the SMART group outperforming the Memory training group.

For ability to provide interpretive statements about the texts, results indicated a significant improvement for the SMART group [*t*(9) = −2.372, *p* = 0.042, *d* = 0.750], along with no significant gain demonstrated for the Memory group [*t*(9) = −1.718, *p* = 0.120, *d* = 0.543]. However, ANCOVA results indicated no significant group effect [*F*(1,20) = 0.50, *p* = 0.487, *d* = 0.316].

For ability to recall details from the texts (based on responses to probe questions), the SMART group again demonstrated a significant gain post-training [*t*(9) = −2.423, *p* = 0.038, *d* = 0.766]. The Memory group demonstrated improvement, which was approaching significance, indicating a trend [*t*(9) = −1.975, *p* = 0.084, *d* = 0.658]. ANCOVA results revealed an interaction between groups, indicating that the variable behaved differently across groups, so a direct comparison could not be done. However, inspection of the group slopes (increase from pre- to post-training) revealed a significantly greater rate of increase in the SMART group relative to the Memory group [*F*(1,19) = 12.78, *p* = 0.003, *d* = 1.643].

#### Secondary outcome measures

For full-scale IQ, paired *t*-test results revealed improvement, which was approaching significance for the SMART group, indicating a trend [*t*(9) = −1.905, *p* = 0.071, *d* = 0.602]. No significant improvement was noted for the Memory group [*t*(9) = −1.014, *p* = 0.337, *d* = 0.321]. ANCOVA results indicated no significant group effect [*F*(1,20) = 1.38, *p* = 0.257, *d* = 0.525]. Additionally, no significant gains were detected on individual subtest scores for verbal (Vocabulary) or non-verbal (Matrix Reasoning) aspects for either group.

In terms of working memory, paired *t*-test results revealed a significant increase in sum of scaled scores for the SMART group [*t*(9) = −2.817, *p* = 0.023, *d* = 0.939]. No significant gain was demonstrated for the Memory group [*t*(9) = −1.257, *p* = 0.240, *d* = 0.398]. ANCOVA results indicated no significant group effect for the sum of scaled scores [*F*(1,19) = 1.35, *p* = 0.262, *d* = 0.534]. However, when breaking the scores down into the individual subtests, ANCOVA results for the Letter–Number Sequencing subtest revealed an interaction, and inspection of the group slopes (increase from pre- to post-training) revealed a significantly greater rate of increase in the SMART group relative to the Memory group [*F*(1,19) = 10.84, *p* = 0.005, *d* = 1.513].

For the inhibition task, paired *t*-tests results indicated significantly improved performance for the SMART group [*t*(9) = −2.298, *p* = 0.047, *d* = 0.727]. The Memory group did not demonstrate significant improvement [*t*(9) = −1.628, *p* = 0.138, *d* = 0.515]. ANCOVA results indicated a significant effect of group [*F*(1,20) = 6.80, *p* = 0.018, *d* = 1.166], with the SMART group outperforming the Memory training group.

The results from the parent ratings of real-life executive function behaviors (BRIEF) suggested improvements in both training groups. It should be noted that two questionnaires were not returned at the post-training time point for the SMART group as well as one missing in the Memory group. The SMART group demonstrated improvement approaching significance [*t*(9) = 2.332, *p* = 0.052, *d* = 0.824], indicating a trend. Parent ratings for the Memory group demonstrated significant improvement [*t*(9) = 3.469, *p* = 0.008, *d* = 1.156]. ANCOVA results indicated no significant group effect [*F*(1,17) = 0.06, *p* = 0.805, *d* = 0.119].

## Discussion

The findings from this pilot study suggest that top-down gist reasoning training (SMART) offers a promising protocol to remediate higher-order cognitive deficits in adolescents with TBI. The gains were achieved at chronic stages of recovery, from months to years after sustaining the injury. Gist reasoning training improved abilities to abstract meanings from complex information as well as generalized to specific untrained executive functions of working memory and inhibition. Moreover, findings suggest that top-down modulation of information may also positively impact bottom-up processes (e.g., improved ability to recall important facts). Alternatively, training of rote memorization skills did not yield significant improvements in any of the cognitive performance domains.

These preliminary results are similar to those previously identified in an adult population with TBI (27). Adults with TBI showed significant gains after gist reasoning training in the primary domain of abstracting meaning as well as in related aspects of cognitive function, including memory for text details and executive functions of working memory and inhibition. Finding working memory improvements in conjunction with enhanced gist reasoning is supported by previous work demonstrating a relation between the two abilities in adolescents with TBI (12, 13). Namely, prior evidence indicates a significant contribution of working memory to gist reasoning over and above what is explained by memory for explicit facts (13). Vas et al. (27) additionally observed post-SMART gains in non-verbal reasoning, an aspect which was not found in the current study (as group mean WASI Matrix Reasoning *T*-scores were unchanged). Nonetheless, there was a positive trend for improved full-scale IQ performance for the adolescent TBI SMART group, which includes both verbal and non-verbal reasoning. In general, these findings lend support to the characterization of gist reasoning as a frontally mediated, integrative, top-down process that directs the capacity to encode and retrieve details and involves a complex interplay of cognitive–linguistic processes (23, 25, 29–31, 61, 62).

It should be noted that the observed improvements in ability to abstract complex meanings are not just attributable to practice effects. The results from the Memory training group support this perspective. In contrast to the gist reasoning training group, individuals in the Memory trained group continued to use a “copy and delete” strategy to condense information at post-testing. Rarely did the individuals in the Memory training group transform ideas into novel generalized statements at pre- or post-testing. This pattern adds further support for the test–retest reliability of the TOSL, where individuals perform the same on repeated trials, unless they learn to abstract meanings during processing of incoming information. Thus, repeated exposure alone does not appear to enhance abstraction ability.

Illustrations of directly observed gains in ability to abstract meaning following gist reasoning training are presented in Supplementary Material. Excerpts represent several participant summaries of the final text of the TOSL, a 575-word passage about a man’s life (which reads much like a history lesson). Before training, when asked to provide a high-level summary (i.e., conveying the important information and high-level ideas in their own words), the majority of youth with TBI exhibited limited ability to synthesize information into generalized ideas from the concrete facts. Instead, they tended to simply retell the key ideas in a litany of detail after detail or convey a few basic main ideas (see underlined portions of the “before SMART” excerpts for representative ideas conveyed). Such summaries are representative of lower-level cognitive strategies typical of students at ages 10 and younger that may hinder complex learning required at later developmental stages (13, 15, 37, 63). This surface-level processing of complex information as manifested at baseline testing is reflected in individuals using predominantly a “copy and delete” strategy to condense information during summarization. After SMART, participants manifested significantly improved ability to abstract novel ideas that went beyond the verbatim details. This ability shows they were better able to discern the “bigger picture” or central theme of the text (see underlined portions of the “after SMART” excerpts), which involved integrating concrete details with real-world knowledge.

In terms of spillover gains to real-life function, in their adult TBI study, Vas et al. (27) observed significant improvements for their SMART group in self-reported participation in day-to-day tasks using the Community Integration Questionnaire (CIQ). For the current adolescent TBI study, the preliminary data were mixed. Parents of adolescents in both training groups reported gains after training relative to baseline, particularly in the Memory training group, although group findings should be interpreted with caution due to the presence of several missing data points for the BRIEF at the post-training assessment (questionnaires not returned by the parent) in both groups. Examining functional gains using a parent questionnaire such as the BRIEF can be limited, as it is difficult to mitigate the effects of parent bias, potentially affecting its interpretation. This was a limitation mentioned by the test authors themselves, who stated that there is a possible bias inherent to the fact that rating scales necessitate third-party ratings of a child’s behavior (42). Furthermore, the parents in the current study were enthusiastic about having their teens engage in one-on-one cognitive training, regardless of the specific training protocol they received. At the time of training, none of the students were receiving services elsewhere to remediate ongoing cognitive struggles. The comparable gain in positive parental perspective between groups supports the study design of utilizing an active control group while also highlighting the need for future trials to consider the impact of placebo effects. Ideally, a combination of both direct, naturalistic executive function measures and questionnaire measures of real-life executive function profiles from multiple observers (i.e., including additions such as the BRIEF Teacher or Self-Report measures) could be implemented to more reliably characterize functional benefits of the trainings.

## Limitations and Future Directions

We acknowledge that due to the small-sample size and relative heterogeneity (e.g., wide range of time post-injury) in this preliminary study, firm conclusions cannot be drawn, as these findings must be replicated in a larger trial. However, several of the reported effect sizes are large, and it is likely that the inclusion of more participants would result in additional significant differences for the conditions that currently suggested only a trend, such as in fact recall for the Memory training group. Additionally, the small-sample size precluded analysis of potential interactions, such as the effects of injury severity, age at injury, or time since injury, as well as contributions of other cognitive factors, which could be addressed within a larger study. Another limitation of the study was that the first author administered all of the cognitive training, which is a potential source of experimenter bias. As such, the results should be interpreted with some degree of caution. Due to the clinical expertise necessary for administering each of these novel training programs with this population, for the purposes of this initial pilot study, focus was put on assuring that differences in outcome could not be attributed to having one trainer versus another (i.e., skill level, experience, rapport). Future trials should include aspects that can ensure fidelity of the training among multiple trainers while minimizing potential bias, such as third-party evaluations of video-recorded sessions and participant manipulation checks.

Despite these limitations, we find the strength of the current findings to be encouraging for conducting an expanded trial. Future investigations should also include long-term follow-up assessments (e.g., at 6 months and 1 year post-training) to evaluate maintenance of gains and/or the potential benefit of periodic “booster” sessions, particularly at key academic transitions. In order to explore the convergence of training-induced brain changes and potential repair with concomitant cognitive gains in the pediatric TBI population, further study could also incorporate functional imaging measures before and after training [e.g., Ref. (30, 31, 62)].

## Conclusion

Advancing long-term recovery in TBI, particularly for those still in development, is an essential aim, given that emerging or persistent higher-order deficits can have a detrimental impact on daily-life aspects such as academic performance and social functioning. The preliminary data suggest that traditional cognitive intervention models may need to be extended to later-stage training opportunities beyond the typical rehabilitation time for adolescents with TBI to achieve higher levels of cognitive performance at older ages. Higher-order cognitive training can play an important clinical role in addressing needs of the many young individuals with brain injury who demonstrate near normal general intellectual functioning and linguistic skills yet experience persistent difficulties on functional tasks that necessitate synthesizing large amounts of information. Currently, protocols for training higher-order top-down cognitive processes are rare and, thus, less likely to be targeted for treatment (64, 65). By taking a top-down, strategy-based approach (e.g., gist reasoning training such as SMART), not only can gains be realized in higher-order functions such as reasoning and abstract thinking, but benefits are likely to spill over to other supportive cognitive functions such as working memory and inhibition, hallmark deficits in TBI. Perhaps even more intriguing is that the gains were achieved after only 6 h of training, so the cost–benefit of this type of top-down approach could be tremendous. For example, if 6 h of training increases the academic and workplace productivity of an individual, reliance on external supports could be decreased and quality of life improved in effect. Ultimately, training of gist reasoning strategies as a routine way to process any complex information has the potential to not only empower young people with brain injury to effectively combat “information overload,” but also promote overall cognitive health and optimize brain repair across their lifespan. The hope is to combat the detrimental alternative of stalled cognitive recovery corresponding with the TBI being managed primarily as an acute problem, with no treatment delivered at chronic stages when significant gains are still possible.

## Conflict of Interest Statement

The authors declare that the research was conducted in the absence of any commercial or financial relationships that could be construed as a potential conflict of interest.

## Supplementary Material

The Supplementary Material for this article can be found online at http://www.frontiersin.org/Journal/10.3389/fneur.2014.00087/abstract
