# Supplementary material for: Cognitive Gains from Gist Reasoning Training in Adolescents with Chronic-Stage Traumatic Brain Injury
Source: Front Neurol. 2014 Jun 11;5:87. doi: 10.3389/fneur.2014.00087 (PMC4052737; doi:10.3389/fneur.2014.00087)
Supplement: Supplementary file 1 [file Data_Sheet1.DOCX]

Supplementary Material. *Summary Excerpts from Before and After Gist Reasoning Training (SMART)*

| Before SMART: underlined portions indicate representative details or basic main ideas conveyed |
| --- |
| - John graduated from college. He became a teacher but was too easy on his students. Then he became a lawyer and was too soft with his clients and wouldn’t take high paying cases. He then opened a store but his prices were too low. He tried to be a minister but his thoughts on slavery weren’t right at the time. During the war he volunteered in a group but he had to resign because of his health. For the last five years of his life he worked as a clerk. - John Pierpont was a man that had a hard time finding a job that he liked all his life, so he died as a failure, but he is remembered for the song Jingle Bells. - He lived his whole life and died thinking that he was a failure and could never finish anything that he started. Little did John know he wasn't a failure. Now John is seen as a very successful man that changed lots of ways that needed to be changed, like the law, credit, slavery, and many more things. |
| After SMART: underlined portions indicate representative abstracted, novel ideas or central themes conveyed |
| - John Pierpont failed at every job he tried because he was too generous. When he died, he felt as though he accomplished nothing. When in reality, he led an incredible life of remembered generosity. - John Pierpont was a very socially involved person. - He gave our society the gift of well service to one another. With his kind heart, others benefited from him and learned from him, which is the best gift of all. - He became a good man, endowed society. - John Pierpont left the world with a gift of service and how important it is to give back to your community. |
